# Proteomic Profiling of a Mouse Model for Ovarian Granulosa Cell Tumor Identifies VCP as a Highly Sensitive Serum Tumor Marker in Several Human Cancers

**DOI:** 10.1371/journal.pone.0042470

**Published:** 2012-08-01

**Authors:** Marie-Noëlle Laguë, Raphaëlle Romieu-Mourez, Éric Bonneil, Alexandre Boyer, Nicolas Pouletty, Anne-Marie Mes-Masson, Pierre Thibault, Marie-Ève Nadeau, Derek Boerboom

**Affiliations:** 1 Centre de Recherche en Reproduction Animale, Faculté de Médecine Vétérinaire, Université de Montréal, Montréal, Québec, Canada; 2 Lady Sir Mortimer B. Davis Jewish General Hospital & Lady Davis Institute for Medical Research, Montréal, Québec, Canada; 3 Institut de Recherche sur le Cancer et en Immunologie, Université de Montreal, Montréal, Québec, Canada; 4 Département de Pathologie et Microbiologie, Faculté de Médecine Vétérinaire, Université de Montréal, Montréal, Québec, Canada; 5 Centre de recherche du Centre Hospitalier de l'Université de Montréal CRCHUM and Institut du cancer de Montréal, Montréal, Québec, Canada; 6 Département de Sciences Cliniques, Faculté de Médecine Vétérinaire, Université de Montréal, Montréal, Québec, Canada; Vanderbilt University Medical Center, United States of America

## Abstract

The initial aim of this study was to identify novel serum diagnostic markers for the human ovarian granulosa cell tumor (GCT), a tumor that represents up to 5% of all ovarian cancers. To circumvent the paucity of human tissues available for analyses, we used the *Ctnnb1* ^tm1Mmt/+^;*Pten* ^tm1Hwu/tmiHwu^;*Amhr2* ^tm3(cre)Bhr/+^ transgenic mouse model, which features the constitutive activation of CTNNB1 signaling combined with the loss of *Pten* in granulosa cells and develops GCTs that mimic aggressive forms of the human disease. Proteomic profiling by mass spectrometry showed that vinculin, enolase 1, several heat shock proteins, and valosin containing protein (VCP) were more abundantly secreted by cultured mouse GCT cells compared to primary cultured GC. Among these proteins, only VCP was present in significantly increased levels in the preoperative serum of GCT cancer patients compared to normal subjects. To determine the specificity of VCP, serum levels were also measured in ovarian carcinoma, non-Hodgkin's lymphoma and breast, colon, pancreatic, lung, and prostate cancer patients. Increased serum VCP levels were observed in the majority of cancer cases, with the exception of patients with lung or prostate cancer. Moreover, serum VCP levels were increased in some GCT, ovarian carcinoma, breast cancer, and colon cancer patients who did not otherwise display increased levels of widely used serum tumor markers for their cancer type (e.g. inhibin A, inhibin B, CA125, CEA, or CA15.3). These results demonstrate the potential use of VCP as highly sensitive serum marker for GCT as well as several other human cancers.

## Introduction

Serum markers are of considerable value for the clinical screening, diagnosis, and follow-up of cancers. An ideal marker should have high sensitivity and/or high specificity, in order to discriminate cancer patients from healthy subjects as well as from patients with benign tumors or unrelated conditions. Most currently used serum markers are hormones, glycoproteins, or other proteins overexpressed by cancer cells. These markers are usually not specific to a unique cancer type and sometimes lack sensitivity [1]. Several strategies have been recently proposed to identify new serum tumor markers [2]. Differential proteomic analyses of serum specimens from cancer patients and healthy subjects are approaches of choice, but are hampered by the paucity of material in rare cancers as well as by the difficulty in detecting proteins that are expressed at low levels compared to abundant normal serum proteins. An alternative two-step approach involves the initial identification of proteins that are differentially expressed and/or secreted between normal and tumor cells, followed by the identification of these proteins in the serum of cancer patients. This approach ideally requires the isolation of primary tumor cells and corresponding normal cells. In this context, the use of relevant animal models of tumor development can provide essential starting materials for proteomic or genomic analysis, and lead to the identification of tumor-specific candidate proteins or genes whose expression can be then be investigated in human samples, as demonstrated in ovarian cancer [3], [4], [5].

The ovarian granulosa cell tumor (GCT) is the most prevalent of the sex cord/stromal subgroup of ovarian tumors in women, and is thought to represent up to 5% of all ovarian cancers. Over the past few years, our group has focused on the elucidation of the molecular etiology of human GCT, as well as on the creation of relevant animal GCT models. We found evidence that misregulation of both the WNT/CTNNB1 (ß-catenin) and PI3K/AKT signaling pathways occur in human GCT [6], [7]. Transgenic mice with constitutive activation of CTNNB1 in granulosa cells (GC, *Ctnnb1* ^tm1Mmt/+^;*Amhr2* ^tm3(cre)Bhr/+^) develop precancerous ovarian lesions that often progress into GCT later in life [6]. The loss of the PI3K/AKT signaling antagonist gene *Pten* in GC rarely causes GCT, but concomitant activation of CTNNB1 and loss of *Pten* in the *Ctnnb1* ^tm1Mmt/+^;*Pten* ^tm1Hwu/tm1Hwu^;*Amhr2* ^tm3(cre)Bhr/+^ (CPA) model results in the development of aggressive, metastatic GCT with 100% penetrance [7]. We have proposed that the CPA mouse is the best model currently available for the analysis of GCT biology as well as for preclinical animal studies aimed at developing novel therapeutic interventions and/or diagnostic tools.

We hypothesized that the CPA mouse model could be used for the identification of differentially expressed, GCT-associated proteins, and that these would translate to clinically useful novel serum diagnostic markers of the human disease. Here we present a differential secretome analysis comparing primary cultured GC from normal mice to GCT cells from CPA mice. This approach led to the identification of vasolin containing protein (VCP) as a potentially clinically relevant serum marker for human GCT, as well as for other forms of cancer.

## Results

### Identification of proteins secreted selectively in mouse GCT

**Figure 1:**
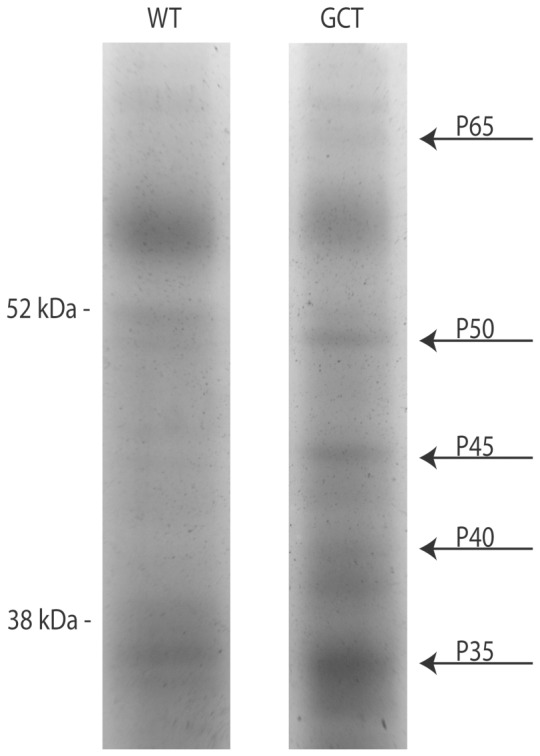
Differential protein expression in mouse granulosa cell and GCT cell culture media. Samples were separated by 10% SDS-PAGE followed by silver staining. Arrows indicate examples of proteins expressed selectively in the GCT cell culture media, along with approximate molecular weights. A total of 3 gels with different acrylamide contents were studied and 14 bands (with proteins from 12 to 116 KDa) were subjected to mass spectrometry analysis. Only one of the 3 gels is shown on the figure.

Proteomic profiling was used with the objective of identifying secreted proteins that could represent new serum diagnostic markers specific for GCT. Spent culture media were obtained from cultured GCT cells from CPA mice or from normal GC from eCG-stimulated ovaries. Proteins from the media were separated by SDS-PAGE and fourteen protein bands observed in GCT but not in GC medium were subjected to mass spectrometry analysis (Figure 1). This process identified a number of proteins that were more abundantly secreted or shed by GCT cells relative to normal GC (Table 1). VCL, ENO1, several heat shock proteins (HSPA8/HSC70, the constitutive and inducible HSP90 alpha isoforms HSP90ab1 and HSP90aa1, and HSPA4), and VCP were the most consistently identified proteins from the GCT secretome.

**Table 1:** Mass spectrometry analysis of the secretome of mouse GCT from CPA transgenic mice.

Protein Identification	Protein Description	Protein Score	Peptides
**IPI00405227**	**Vcl Vinculin**	**746**	**21**
IPI00323357	Hspa8 Heat shock protein 8/Hsc70	488	27
**IPI00554929**	**Hsp90ab1 Heat shock protein 90 alpha (cytosolic), class B member 1**	**468**	**13**
**IPI00622235**	**Vcp Valosin containing protein/p97**	**372**	**12**
IPI00462072	Eno1 Enolase 1 alpha non-neuron	349	9
**IPI00330804**	**Hsp90aa1 Heat shock protein 90, alpha (cytosolic), class A member 1**	**346**	**10**
IPI00110827	Acta1 Actin alpha 1 skeletal muscle	228	9
IPI00124707	Fstl1 Follistatin-like 1	228	5
IPI00117312	Got2 Glutamate oxaloacetate transaminase 2, mitochondrial	224	4
**IPI00331556**	**Hspa4 Heat shock protein 4**	**201**	**5**
IPI00466069	Eef2 Eukaryotic translation elongation factor 2	178	5
IPI00135231	Idh1 Isocitrate dehydrogenase 1 (NADP+), soluble	171	4
IPI00223231	Qsox1 quiescin Q6 sulfhydryl oxidase 1	157	4
**IPI00126343**	**Sparc Secreted acidic cysteine rich glycoprotein**	**132**	**4**
IPI00313900	Lum Lumican	119	3
**IPI00113517**	**Ctsb Cathepsin B**	**113**	**3**
IPI00133208	Hspa1a Heat shock protein 1A/Hsp70-3	106	3
IPI00221402	Aldoa Aldolase A, fructose-bisphosphate	105	7
IPI00228548	Eno3 Enolase 3 beta muscle	101	3
**IPI00124441**	**Wif1 Wnt inhibitory factor 1**	**82**	**2**
**IPI00331286**	**B2m beta-2-microglobulin**	**79**	**3**
**IPI00113863**	**Timp2 Tissue inhibitor of metalloproteinase 2**	**79**	**2**
IPI00313296	Rnh1 Ribonuclease/angiogenin inhibitor 1	78	2
IPI00762198	Hbb-b1 Hemoglobin, beta adult major chain	66	2
IPI00109061	Tubb2b Tubulin beta-2B	63	2
IPI00109073	Tubb4 Tubulin beta-4	63	2
IPI00131547	Serpine1 Serine (or cysteine) peptidase inhibitor, clade E, member 1	55	2
IPI00130391	Prss1 Protease, serine, 1	54	2
IPI00407502	C1r Complement C1r-A subcomponent precursor	50	2
IPI00114209	Glud1 Glutamate dehydrogenase 1	49	3
IPI00127407	Plod1 Procollagen-lysine, 2-oxoglutarate 5-dioxygenase 1	43	2
IPI00119809	Lgals3bp Lectin, galactoside-binding, soluble, 3 binding protein	39	2
IPI00122528	Tgfbi Transforming growth factor-beta-induced	31	2

Protein identification (International Protein Index identifier) and protein description are given along with the overall score and the number of peptides identified by mass spectrometry as described in Materials & Methods. Proteins in bold were studied further.

### VCP is a serum marker for GCT and other cancer types

**Figure 2:**
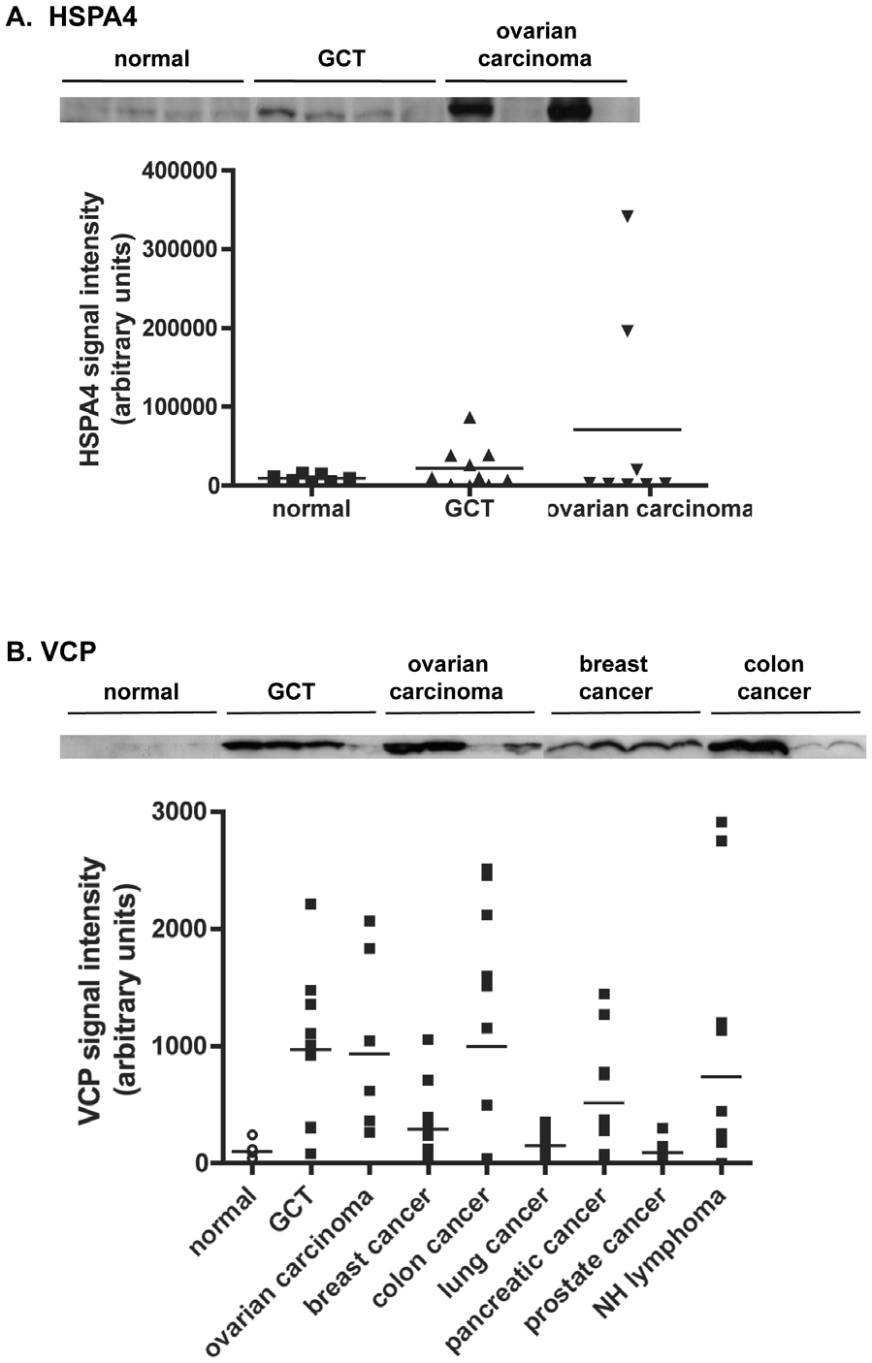
Serum levels of HSPA4 and VCP in healthy volunteers and in cancer patients. (A) HSPA4 levels in the serum of women with GCT or ovarian carcinoma. Equal amounts of serum protein were separated by SDS-PAGE and were subjected to immunoblot analysis for HSPA4 levels (representative blots are shown in the top panel, each lane represents a single donor). Densitometry analyses of signals obtained with the HSPA4 immunoblot analyses are reported in a graph in which each dot represents a single donor (bottom, horizontal bar = mean). No significant difference in HSPA4 levels was detected among groups by one-way ANOVA. (B) VCP levels are increased in the serum of cancer patients. Sera were analyzed as in (A) for the presence of VCP in patients with breast, colon, lung, pancreatic or prostate cancer, ovarian carcinoma, GCT, or non-Hodgkin's (NH) lymphoma. Statistically significant differences (*P*<0.05) were detected between the control (normal) and GCT and colon cancer groups.

We next investigated whether the proteins secreted by GCT cells from CPA mice could serve as serum markers in human GCT patients. Human homologs of nine proteins identified in the secretome analyses (B2M, CTSB, HSPA4/HSP70, HSP90, SPARC, TIMP-2, VCP, VCL, and WIF-1) were selected for further study based on known gene functions and antibody availability. Serum samples were obtained from healthy volunteers and from patients with GCT prior to treatment. No significant differences in the levels of B2M, CTSB, HSP90, SPARC, TIMP-2, VCL or WIF-1 were observed by immunoblot analyses in the serum of GCT patients compared to healthy subjects (data not shown). Marginally increased levels of HSP4A were observed in the serum of some GCT patients compared to normal subjects, however the differences were not statically significant, and deemed unlikely to be clinically useful (Figure 2A). VCP levels were low in immunoblot analyses of serum samples from healthy subjects, but were significantly increased in the majority of serum samples from GCT patients (*P*<0.05). With a reference value for VCP levels set at the mean of immunoblot band intensity values of the healthy controls plus twice the standard deviation, we observed that 8 out of 9 women with GCT displayed increased serum VCP levels (Figure 2B, Table 2). To determine the specificity of VCP as a tumor marker for GCT, serum VCP levels were assessed in patients with ovarian carcinomas, as well as in small cohorts of patients with major non-ovarian cancers of known histology and grade (Table 3). Surprisingly, elevated serum VCP levels were detected in clinically significant proportions of patients with ovarian carcinoma (8 of 8), breast cancer (5 of 12), colon cancer (7 of 12), pancreatic cancer (8 of 12) or non-Hodgkin's lymphoma (5 of 12) (Figure 2B and Table 3). However, serum VCP levels were not meaningfully increased in patients with lung or prostate cancer.

**Table 2:** Serum levels of GCT markers in healthy women or in women with GCT.

Group	Diagnosis	Menopausal status	INHA (ng/L)	INHB (ng/L)	CA-125 (U/ml)	VCP (arbitary units)
control		Post	NE	<20.0	6	110
control		Pre	NE	<20.0	6	46
control		Post	NE	<20.0	6	121
control		Post	NE	**24.7**	9	0
control		Pre	NE	<20.0	20	241
control		Post	NE	<20.0	7	95
control		Post	NE	<20.0	14	117
GCT	Adult GCT	Pre	<13	**26.7**	NE	**1010**
GCT	Adult GCT	Pre	**92**	**>1136.0**	NE	**921**
GCT	Adult GCT: Relapse	Post	<13	**63.8**	NE	**1110**
GCT	Adult GCT	Pre	<13	<20.0	NE	**1478**
GCT	Adult GCT	Pre	**28.1**	**59.7**	**57**	**2212**
GCT	Adult GCT	Post	**213.5**	**>1136.0**	**231**	**1357**
GCT	Adult GCT	Pre	**145.1**	**>1136.0**	**120**	**964**
GCT	Adult GCT	Pre	**21.1**	<20.0	31	**308**
GCT	Adult GCT	Post	<13	**391**	NE	83

Measurements that exceeded the normal reference range are indicated in bold. For inhibin A and B (INHA and INHB), values beneath the detection thresholds by ELISA were defined as normal. For VCP, the reference value was set as the mean of healthy control band intensity in immunoblot analyses+2SD (253 arbitrary units). Note that serum inhibin usually becomes undetectable after menopause in healthy women. Interpretation of premenopausal inhibin values can be difficult due to their secretion both by growing ovarian follicles and by GCTs. NE: not evaluated.

**Table 3:** Tumor clinical features and VCP serum level in tested cancer patients.

	Sample Number	Histology	Grade/Differentiation	VCP levels
**Healthy donor**				
	1			110.0
	2			46.0
	3			121.0
	4			0.0
	5			240.9
	6			94.8
	7			116.6
**GCT**				
	AM3	Adult GCT	IA	**1010.4**
	AM4	Adult GCT	IA	**921.1**
	AM5	Adult GCT: Relapse	X	**1109.6**
	AM6	Adult GCT	IA	**1478.0**
	AM7	Adult GCT	IIIC	**2212.0**
	AM8	Adult GCT	IA	**1357.0**
	AM9	Adult GCT	IA	**964.0**
	AM10	Adult GCT	IC	**308.0**
	AM11	Adult GCT	IIIc	83.0
**ovarian cancer**				
	AM12	CL adenocarcinoma	IC	**362.0**
	AM13	CL adenocarcinoma	IIB	**266.0**
	AM14	EM adenocarcinoma	I	**2069.0**
	AM15	EM adenocarcinoma	IIIC	**1048.0**
	AM16	mucinous cystadenocarcinoma	IA	**261.6**
	AM17	mucinous cystadenocarcinoma	IIIC	**618.0**
	AM18	serous cystadenocarcinoma	IIIC	**1046.4**
	AM19	serous cystadenocarcinoma	III	**1836.7**
**breast cancer**				
	B00404105	invasive mammary NOS	III	121.8
	B00405105	invasive mammary NOS	I	**708.8**
	B00515113	invasive mammary NOS	III	**394.8**
	B00516111	invasive mammary NOS	II	**343.9**
	B00596105	invasive mammary NOS	II	126.7
	B00649114	invasive mammary NOS	II	237.6
	F00020105	IDC	I	65.2
	F00048105	IDC	II	72.3
	F00049105	IDC	III	**1060.0**
	F00116105	IDC	II	30.9
	F00117105	IDC	III	**351.1**
	F00372103	IDC	II	25.4
**colon cancer**				
	B00266111	Adenocarcinoma	II	**2452.8**
	B00279112	Adenocarcinoma	II	**2510.2**
	B00443114	Adenocarcinoma	II	8.1
	B00457113	Adenocarcinoma	II	2.6
	B00502102	Adenocarcinoma	IV	11.3
	B00530114	Adenocarcinoma	II	31.8
	B00674115	Adenocarcinoma	II	**1157.1**
	B00703104	Adenocarcinoma	IV	**1513.5**
	B00728115	Adenocarcinoma	IV	44.6
	B01057112	Adenocarcinoma	IV	**1595.2**
	B01157113	Adenocarcinoma	IV	**2120.2**
	B01595113	Adenocarcinoma	IV	**498.3**
**pancreatic cancer**				
	A01411102	invasive ductal adenocarcinoma	III	4.23
	B00537112	Adenocarcinoma	III	0
	B00627112	Adenocarcinoma	X	**367.56**
	B02973106	Ductal adenocarcinoma, NOS	II	**277.7**
	D00086102	ductal adenocarcinoma	II	**756.32**
	D00205104	adenocarcinoma	III	**1445.88**
	D00408101	adenocarcinoma	X	**1268.59**
	D00544102	adenocarcinoma	II	**372.54**
	D01000101	ductal carcinoma	II	**779.26**
	D01013101	ductal carcinoma	II	**761.58**
	E01584101	invasive ductal adenocarcinoma	II	72.89
	F00302101	ductal adenocarcinoma	II	77.15
**lung cancer**				
	A00195101	adenocarcinoma	III	41.58
	A00242102	LC undifferentiated carcinoma	X	158.4
	A00327102	adenocarcinoma	I	195.6
	A00392101	adenocarcinoma	I	148.76
	A00404102	squamous carcinoma	II	86.63
	A00699101	adenocarcinoma	X	**272.46**
	A00710101	adenocarcinoma	III	168.24
	A00728102	adenocarcinoma	I	208.09
	A01015104	LC undifferentiated carcinoma	X	14.4
	A01341101	squamous carcinoma	III	82.37
	A01429101	squamous carcinoma	II	**351.27**
	A02117112	squamous carcinoma	II	119.04
**prostate cancer**				
	A01598101	adenocarcinoma	T3b	89.97
	A01738101	adenocarcinoma	T3b	44.24
	B02131103	adenocarcinoma	T3b	42.92
	B02408102	adenocarcinoma	T2c	**298**
	B02409102	adenocarcinoma	T2c	114.31
	B02469101	adenocarcinoma	T3,NOS	11.07
	B02682102	adenocarcinoma	T2c	147.48
	B02683103	adenocarcinoma	T2c	124.46
	B02704103	adenocarcinoma	T2c	135.62
	B02725101	adenocarcinoma	T2c	3.72
	B03083102	adenocarcinoma	T3b	37.73
	F00339102	adenocarcinoma	T3a	34.9
**NHDG**				
	A00730101	mixed; follicular and diffuse large B cell lymphoma	IE	0
	A01749102	diffuse large B cell lymphoma	X	0
	B01423113	small bowel lymphoma NOS	IVE	**2753.45**
	B01563111	testicular lymphoma NOS	IV	181.93
	B01824113	follicular lymphoma	IV	0
	B02332112	mixed; follicular and diffuse large B cell lymphoma	III	252.9
	B02337111	mixed; follicular and diffuse large B cell lymphoma	IV	0
	D00776101	mixed; follicular and diffuse large B cell lymphoma	I	**1203.38**
	D02063102	follicular lymphoma	X	**2911.73**
	D02342103	marginal zone lymphoma	X	**447.32**
	D02482102	NA	X	**1136.11**
	E00273103	follicular lymphoma	IV	0

For VCP, the reference value was set as the mean of healthy control band intensity in immunoblot analyses+2SD (253 arbitrary units). Positive VCP values are indicated in bold. X: unknown grade/differentiation, CL: clear cell, EM: endometroid, NOS: not otherwise specified, IDC: invasive ductal carcinoma, LC: large cell, NHDG: non-Hodgkin's lymphoma, NA: not available.

### Specificity and sensitivity of VCP relative to widely-used serum diagnostic markers

**Figure 3:**
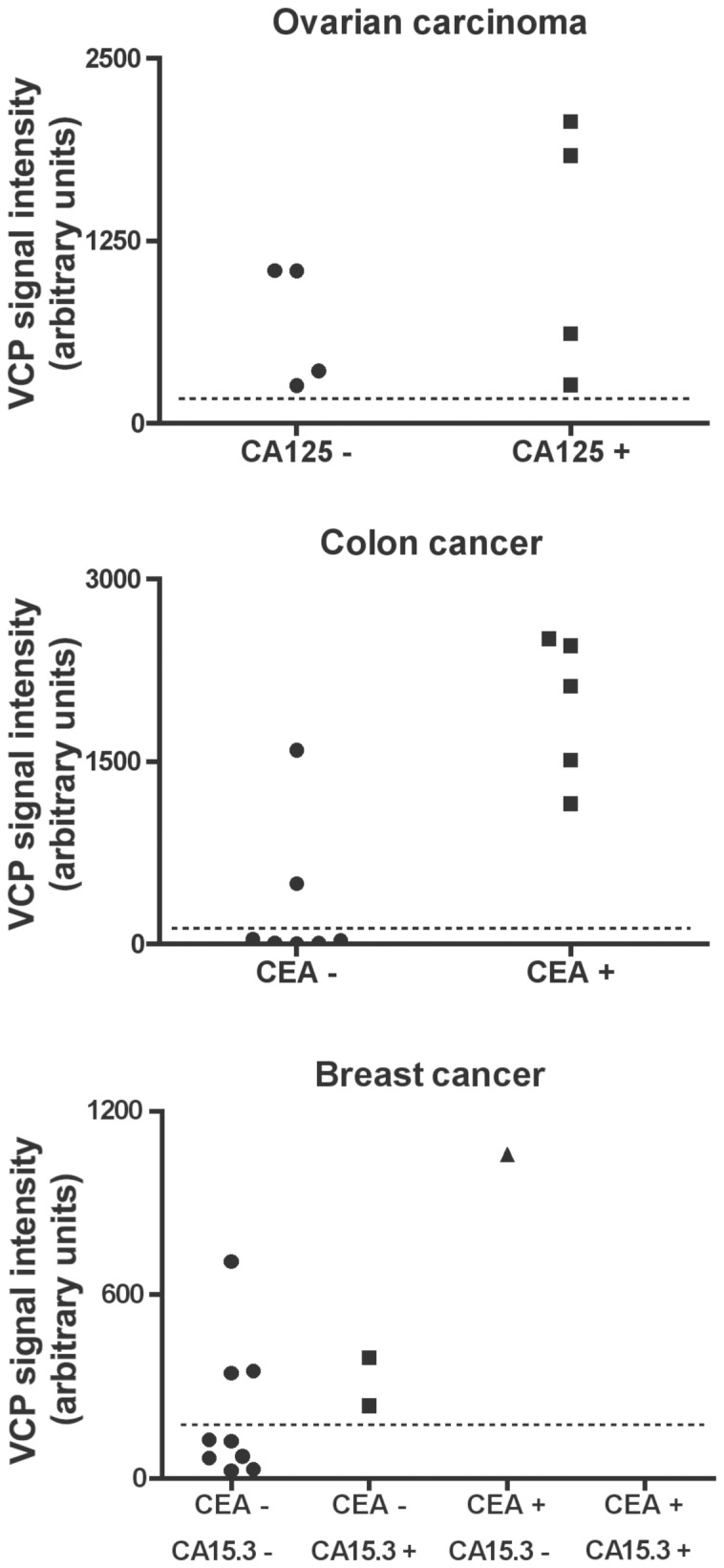
Assessment of serum levels of VCP compared to serum tumor markers currently used for ovarian carcinoma, colon cancer, and breast cancer. Sera from patients were tested for VCP levels by immunoblot analyses and the dotted line shows VCP cutoff values established in healthy donors. In addition, sera were tested by ELISA for the presence (+) or absence (−) of increased levels of CA125 in ovarian carcinoma, CEA in colon cancer, or CEA and CA15.3 in breast cancer. The normal ranges of CA125, CEA, and CA15.3 are below 35 U/ml, 7 µg/l, and 29 kU/l, respectively.

We next compared the relevancy of VCP to that of other commonly-used serum diagnostic markers for various cancer types. Presently, the most useful serum markers for GCT in postmenopausal women are inhibin A and inhibin B [8]. In our cohort, inhibin A and inhibin B serum levels were increased in 5 out 9 and 7 out of 9 of patients with GCT (Table 2), respectively. There was no clear correlation between preoperative levels of inhibin A, inhibin B and VCP in GCT patients, nevertheless one patient had undetectable serum levels of inhibin A and inhibin B but highly increased levels of VCP (Table 2). The sensitivity of VCP therefore appears to compare favorably to that of the inhibins, and VCP could serve to identify rare inhibin-negative GCT patients. Serum CA125 is increased in approximately 50% of women with early ovarian carcinoma and in over 80% of women with advanced disease and is useful for monitoring therapy [9]. In our small ovarian carcinoma cohort, 4 of 8 patients tested positive for CA125, whereas VCP measurement detected cancer in all patients, including those negative for CA125 (Figure 3). CEA is the most widely used serum tumor marker for colon cancer. Serum CEA is elevated in less than 25% of early stage colon cancer and 75% of late-stage cancer and is useful for determining prognosis, monitoring therapy and surveillance [10]. In the 12 colon cancer patients that we tested, 5 were positive for CEA. VCP measurement detected cancer in all CEA-positive patients, in addition to two that were CEA-negative (Figure 3). CEA and CA15.3 are the most commonly used serum markers for breast cancer. Assessment of these markers is not recommended for prognosis but rather for postoperative follow-up as well as monitoring in advanced diseases [10]. In the 12-case cohort that we examined, 3 patients tested positive for either CEA or CA15.3, with the remainder negative for both. VCP levels were elevated in all three patients that were CEA- or CA15.3-positive, and also in three patients that were negative for both markers (Figure 3). Thus, VCP serum levels were significantly increased in the majority of the tested cancer patients, including in some otherwise negative for established serum tumor markers.

## Discussion

The VCP protein is a AAA+ ATPase associated with diverse cellular activities. VCP is necessary for the maintenance of cellular protein homeostasis and regulates the expression of proteins involved in many functions such as DNA replication, mitosis, protein degradation, endocytosis, membrane fusion, and organelle biogenesis. Specifically, VCP acts on ubiquitinated substrates molecules and regulates protein turnover by balancing the proteosomal degradation of soluble proteins or misfolded protein aggregates localized in the ER lumen or the cytosol. Depending on conformational recoverability of complexes with VCP and target protein substrates, VCP either promotes their degradation, segregates them from large protein complexes, or modulates their ubiquitination by competing ubiquitin conjugation and deconjugation machineries (reviewed in [11]). VCP associates with numerous ubiquinated substrates, and the functions of VCP in different cell types relates in part to the specific tissue expression of substrates and co-factors. In neurons and muscle cells, VCP interaction with NF-1, UNC45b, and caveolin-3 regulates synaptogenesis and myofibrogenesis (reviewed in [12]). Some autosomal dominant mutations in the VCP gene cause inclusion body myopathy with Paget disease of the bone and frontotemporal dementia (IBMPFD) which is a rare, late age–onset inherited degenerative disorder that can affect the muscles, bones and brain [13]. In addition to essential functions in homeostasis, VCP was shown to regulate the half-life and levels of several proteins with cancer-modulating functions. For instance, VCP in cooperation with several ubiquitin ligases regulates the turn-over of IkappaB [14], HIF-1 [15], p53 [16], or ErbB3 [17]. Mutations and/or misregulation of VCP functions in cancer cells are still not well characterized, as are their possible causative effects in tumorigenesis. Nevertheless, overexpression of VCP has recently been evidenced *in situ* in a wide array of human cancer types, and analyses of large patient cohorts demonstrated significantly increased expression levels of VCP by tumor cells often correlate with disease progression [18], [19], [20], [21], [22], [23], [24], [25]. However, the present report is the first to identify increased VCP levels in the serum of cancer patients.

Taken together, our results indicate that VCP represents a highly sensitive, potentially clinically useful serum tumor marker for a variety of human cancers. Although its lack of specificity for a single cancer type suggests that it is unlikely that VCP serum measurement could be used as a screening tool, its sensitivity equivalent or superior to many commonly used markers indicates that it could be used in applications such as monitoring treatment efficacy, or monitoring for disease recurrence. Additionally, VCP could be used for diagnosis in conjunction with more cancer type-specific markers to increase the overall sensitivity of the assay. Screening of much larger cohorts will be required to determine the sensitivity of serum VCP measurement for the detection of different stages of cancer progression, and of different cancer subtypes. Furthermore, the specificity of VCP with regards to the detection of neoplastic vs non-neoplastic disease will need to be assessed before its usefulness as a tumor marker can be definitively established. As immunoblotting is limited both in terms of quantitativeness and technical practicality, large-scale screening will require the development of a high-throughput ELISA assay for VCP.

In summary, our results demonstrate that proteomic profiling of tumor secretomes from clinically relevant mouse models can lead to the identification of candidate circulating proteins associated with tumorigenesis in humans. We report that VCP is overexpressed in the serum of cancer patients, and that it may represent a new clinically useful marker for cancer detection.

## Materials and Methods

### Mice

*Ctnnb1* ^tm1Mmt/+^;*Pten* ^tm1Hwu/tm1Hwu^;*Amhr2* ^tm3(cre)Bhr/+^ (CPA) transgenic mice were generated as previously described [7] and maintained on a C57BL/6 genetic background. In these mice, constitutive activation of CTNNB1 signaling is due to the deletion of the third exon of *Ctnnb1*, resulting in the production of a dominant-stable CTNNB1 mutant protein that lacks the phosphorylation sites required for its proteosomal degradation [6]. CPA mice develop GCT perinatally and die before 8 weeks of age [7]. All animal procedures were approved by the Institutional Animal Care and Use Committee and were conform to the USPHS Policy on Humane Care and Use of Laboratory Animals.

### Cell culture

Normal GC were isolated using the method described by Zeleznik et al. [26]. Briefly, immature (23–26 day-old) C57BL/6 mice were injected I.P. with 5 IU equine chorionic gonadotropin (eCG, Folligon, Intervet, Schering-Plough) to induce follicular growth. Forty-eight hours later, animals were sacrificed and the ovaries punctured with a 25-gauge needle to free GCs into the medium (0.9% NaCl). The cell suspension was centrifuged at 1,000 *g* for 5 min and resuspended GC were plated into 24 well plates at 70% confluency in DMEM/F12 medium (Sigma Aldrich) supplemented with 5% FBS (Invitrogen), penicillin and streptomycin (P/S). GCT from 21 to 25 day-old CPA mice were excised, minced with a size 10 scalpel blade and digested for 2 h with 0.1% collagenase from *Clostridium histolyticum* (Sigma) in serum-free DMEM with P/S. Cells were then centrifuged at 1,000 *g* for 10 min and resuspended in DMEM supplemented with 10% FBS and P/S prior to plating into 100 mm cell culture dishes (25×10^6^ cells/dish).

### Differential secretome analyses

Twenty-four hours after plating, cultured GC and GCT cells were washed with HBSS (Invitrogen) and incubated for 24 h in serum-free DMEM. Supernatants were collected and concentrated 80-fold using Amicon Ultra-4 centrifugal filter units (Millipore) with a 3 kDa molecular weight cut-off. Protein concentrations were quantified using the method of Bradford (Bio-Rad protein assay) and 4–6 µg protein samples were separated by SDS-PAGE. Following silver staining, proteins bands found in GCT but not in GC samples (n = 14) were excised from the gel. The gel slices were destained with 50% methanol then reduced in 10 mM DTT for 1 hour at 56°C and alkylated in 55 mM chloroacetamide for one hour at room temperature. After washing in 50 mM ammonium bicarbonate, the gel pieces were shrunk in 100% acetonitrile (ACN). Digestion was performed using trypsin in 50 mM ammonium bicarbonate for 8 hours at 37°C. The peptides were finally extracted in 90% ACN/0.5 M urea and dried in a speed vacuum. Samples were resolubilized in 5% ACN with 2% formic acid (FA) and separated on a homemade C_18_ column (150 µm×10 cm) using an Eksigent nanoLC-2D system. A 56-min gradient from 5–60% ACN (0.2% FA) was used to elute peptides from a homemade reversed-phase column (150 µm i.d. ×100 mm) with a flow rate set at 600 nanoliter/min. The column was directly connected to a nanoprobe interfaced with an LTQ-Orbitrap Velos mass spectrometer (Thermo-Fisher). Each full MS spectrum was followed by twelve MS/MS spectra (thirteen scan events), where the twelve most abundant multiply-charged ions were selected for MS/MS sequencing. Tandem MS experiments were performed using collision-induced dissociation in the linear ion trap. The data were processed using the 2.1 Mascot (Matrix Science) search engine with tolerance parameters set to 15 ppm and 0.5 Da for the precursor and the fragment ions respectively. The selected variable modifications were carbamidomethyl (C), deamidation (NQ), oxidation (M) and phosphorylation (STY). The selected database was human IPI database v.3.54 with 150858 sequences.

### Serum samples

Serum samples from patients with breast, colon, lung, pancreatic or prostate cancer, or non-Hodgkin's lymphoma (n = 12 for each cancer type) were obtained from the Ontario Tumour Bank. Serum samples from healthy volunteers (n = 7) and patients with GCT (n = 9) or ovarian carcinoma (n = 8; 2 clear cell, 2 serous, 2 mucinous and 2 endometrioid ovarian cancers) were obtained from the Réseau de recherche du cancer du Fonds de recherche Québec - Santé. Procedures were approved by the FRQS-RRCancer Research Committee and the Ontario Cancer Research Ethics Board, as well as the Comité d'éthique de la recherche sur les sujets humains of the Centre hospitalier de l'Université de Montréal. Inhibin A and inhibin B levels were determined by ELISA at the University of Virginia Center for Research in Reproduction Ligand Assay and Analysis Core. Values for inhibin A and B beneath the detection threshold (13 ng/l and 20 ng/l, respectively) were defined as normal. CA15.3 and CEA levels were determined by les Laboratoires du Centre Hospitalier de l'Université de Montréal. Values below 7 µg/l and 29 kU/l for CEA and CA 15.3, respectively, were considered normal. CA125 levels were determined using a commercially available ELISA assay kit and levels were considered as normal when below 35 U/ml (Abnova).

### Immunoblot analyses

Serum samples (4 µl i.e. approximately 200 µg) were separated by SDS-PAGE on 10% acrylamide gels. The gels were transferred to polyvinylidene fluoride membranes (GE Amersham/VWR). Immunodetection was performed with HSPA4- or VCP-specific antibodies (clones ab75977 or ab11433 respectively, Abcam) and horseradish peroxidase-conjugated secondary antibodies (GE Amersham/VWR) and revealed using ECL Detection Reagents (GE Amersham/VWR). Quantification of the protein bands was performed by densitometry analyses using a Kodak Image Station 440CF and Kodak 1D v.3.6.5 software (Eastman Kodak, Rochester, NY). In order to normalize VCP or HSPA4 protein levels between immunoblots, each gel contained two or three common serum samples as references. The reference value for VCP was set as the mean of healthy control band intensities in immunoblot analyses+2SD.

### Statistical analyses

One-way ANOVA with Dunnett's post-test was used to compare VCP levels in healthy women and cancer patients.

## References

[1] Perkins GL, Slater ED, Sanders GK, Prichard JG (2003) Serum tumor markers. Am Fam Physician 68: 1075–1082. 14524394

[2] Alaiya A, Al-Mohanna M, Linder S (2005) Clinical cancer proteomics: promises and pitfalls. J Proteome Res 4: 1213–1222. 16083271 10.1021/pr050149f

[3] Wei BR, Hoover SB, Ross MM, Zhou W, Meani F, et al (2009) Serum S100A6 concentration predicts peritoneal tumor burden in mice with epithelial ovarian cancer and is associated with advanced stage in patients. PLoS One 4: e7670. 19888321 10.1371/journal.pone.0007670 PMC2765613

[4] Pitteri SJ, JeBailey L, Faca VM, Thorpe JD, Silva MA, et al (2009) Integrated proteomic analysis of human cancer cells and plasma from tumor bearing mice for ovarian cancer biomarker discovery. PLoS One 4: e7916. 19936259 10.1371/journal.pone.0007916 PMC2775948

[5] Mullany LK, Fan HY, Liu Z, White LD, Marshall A, et al (2011) Molecular and functional characteristics of ovarian surface epithelial cells transformed by KrasG12D and loss of Pten in a mouse model in vivo. Oncogene 30: 3522–3536. 21423204 10.1038/onc.2011.70 PMC3139785

[6] Boerboom D, Paquet M, Hsieh M, Liu J, Jamin SP, et al (2005) Misregulated Wnt/beta-catenin signaling leads to ovarian granulosa cell tumor development. Cancer Res 65: 9206–9215. 16230381 10.1158/0008-5472.CAN-05-1024

[7] Lague MN, Paquet M, Fan HY, Kaartinen MJ, Chu S, et al (2008) Synergistic effects of Pten loss and WNT/CTNNB1 signaling pathway activation in ovarian granulosa cell tumor development and progression. Carcinogenesis 29: 2062–2072. 18687666 10.1093/carcin/bgn186 PMC2577137

[8] Geerts I, Vergote I, Neven P, Billen J (2009) The role of inhibins B and antimullerian hormone for diagnosis and follow-up of granulosa cell tumors. Int J Gynecol Cancer 19: 847–855. 19574772 10.1111/IGC.0b013e3181a702d1

[9] Carlson KJ, Skates SJ, Singer DE (1994) Screening for ovarian cancer. Ann Intern Med 121: 124–132. 8017726 10.7326/0003-4819-121-2-199407150-00009

[10] Sturgeon CM, Duffy MJ, Stenman UH, Lilja H, Brunner N, et al (2008) National Academy of Clinical Biochemistry laboratory medicine practice guidelines for use of tumor markers in testicular, prostate, colorectal, breast, and ovarian cancers. Clin Chem 54: e11–79. 19042984 10.1373/clinchem.2008.105601

[11] Halawani D, Latterich M (2006) p97: The cell's molecular purgatory? Mol Cell 22: 713–717. 16793541 10.1016/j.molcel.2006.06.003

[12] Weihl CC (2011) Another VCP interactor: NF is enough. J Clin Invest 121: 4627–4630. 22105166 10.1172/JCI61126 PMC3226341

[13] Watts GD, Wymer J, Kovach MJ, Mehta SG, Mumm S, et al (2004) Inclusion body myopathy associated with Paget disease of bone and frontotemporal dementia is caused by mutant valosin-containing protein. Nat Genet 36: 377–381. 15034582 10.1038/ng1332

[14] Dai RM, Chen E, Longo DL, Gorbea CM, Li CC (1998) Involvement of valosin-containing protein, an ATPase Co-purified with IkappaBalpha and 26 S proteasome, in ubiquitin-proteasome-mediated degradation of IkappaBalpha. J Biol Chem 273: 3562–3573. 9452483 10.1074/jbc.273.6.3562

[15] Alexandru G, Graumann J, Smith GT, Kolawa NJ, Fang R, et al (2008) UBXD7 binds multiple ubiquitin ligases and implicates p97 in HIF1alpha turnover. Cell 134: 804–816. 18775313 10.1016/j.cell.2008.06.048 PMC2614663

[16] Valle CW, Min T, Bodas M, Mazur S, Begum S, et al (2011) Critical role of VCP/p97 in the pathogenesis and progression of non-small cell lung carcinoma. PLoS One 6: e29073. 22216170 10.1371/journal.pone.0029073 PMC3245239

[17] Fry WH, Simion C, Sweeney C, Carraway KL 3rd (2011) Quantity control of the ErbB3 receptor tyrosine kinase at the endoplasmic reticulum. Mol Cell Biol 31: 3009–3018. 21576364 10.1128/MCB.05105-11 PMC3133396

[18] Tsujimoto Y, Tomita Y, Hoshida Y, Kono T, Oka T, et al (2004) Elevated expression of valosin-containing protein (p97) is associated with poor prognosis of prostate cancer. Clin Cancer Res 10: 3007–3012. 15131036 10.1158/1078-0432.ccr-03-0191

[19] Yamamoto S, Tomita Y, Hoshida Y, Iizuka N, Kidogami S, et al (2004) Expression level of valosin-containing protein (p97) is associated with prognosis of esophageal carcinoma. Clin Cancer Res 10: 5558–5565. 15328197 10.1158/1078-0432.CCR-0723-03

[20] Yamamoto S, Tomita Y, Hoshida Y, Iizuka N, Monden M, et al (2004) Expression level of valosin-containing protein (p97) is correlated with progression and prognosis of non-small-cell lung carcinoma. Ann Surg Oncol 11: 697–704. 15231524 10.1245/ASO.2004.10.018

[21] Yamamoto S, Tomita Y, Hoshida Y, Nagano H, Dono K, et al (2004) Increased expression of valosin-containing protein (p97) is associated with lymph node metastasis and prognosis of pancreatic ductal adenocarcinoma. Ann Surg Oncol 11: 165–172. 14761919 10.1245/aso.2004.05.012

[22] Yamamoto S, Tomita Y, Nakamori S, Hoshida Y, Iizuka N, et al (2004) Valosin-containing protein (p97) and Ki-67 expression is a useful marker in detecting malignant behavior of pancreatic endocrine neoplasms. Oncology 66: 468–475. 15452376 10.1159/000079501

[23] Yamamoto S, Tomita Y, Nakamori S, Hoshida Y, Nagano H, et al (2003) Elevated expression of valosin-containing protein (p97) in hepatocellular carcinoma is correlated with increased incidence of tumor recurrence. J Clin Oncol 21: 447–452. 12560433 10.1200/JCO.2003.06.068

[24] Yamamoto S, Tomita Y, Uruno T, Hoshida Y, Qiu Y, et al (2005) Increased expression of valosin-containing protein (p97) is correlated with disease recurrence in follicular thyroid cancer. Ann Surg Oncol 12: 925–934. 16189643 10.1245/ASO.2005.07.002

[25] Yamamoto S, Tomita Y, Hoshida Y, Sakon M, Kameyama M, et al (2004) Expression of valosin-containing protein in colorectal carcinomas as a predictor for disease recurrence and prognosis. Clin Cancer Res 10: 651–657. 14760088 10.1158/1078-0432.ccr-1576-03

[26] Zeleznik AJ, Midgley AR Jr, Reichert LE Jr (1974) Granulosa cell maturation in the rat: increased binding of human chorionic gonadotropin following treatment with follicle-stimulating hormone in vivo. Endocrinology 95: 818–825. 4368756 10.1210/endo-95-3-818

